# Rapid Analysis of Glycolytic and Oxidative Substrate Flux of Cancer Cells in a Microplate

**DOI:** 10.1371/journal.pone.0109916

**Published:** 2014-10-31

**Authors:** Lisa S. Pike Winer, Min Wu

**Affiliations:** Seahorse Bioscience Inc., North Billerica, Massachusetts, United States of America; University of Pittsburgh, United States of America

## Abstract

Cancer cells exhibit remarkable alterations in cellular metabolism, particularly in their nutrient substrate preference. We have devised several experimental methods that rapidly analyze the metabolic substrate flux in cancer cells: glycolysis and the oxidation of major fuel substrates glucose, glutamine, and fatty acids. Using the XF Extracellular Flux analyzer, these methods measure, in real-time, the oxygen consumption rate (OCR) and extracellular acidification rate (ECAR) of living cells in a microplate as they respond to substrates and metabolic perturbation agents. In proof-of-principle experiments, we analyzed substrate flux and mitochondrial bioenergetics of two human glioblastoma cell lines, SF188s and SF188f, which were derived from the same parental cell line but proliferate at slow and fast rates, respectively. These analyses led to three interesting observations: 1) both cell lines respired effectively with substantial endogenous substrate respiration; 2) SF188f cells underwent a significant shift from glycolytic to oxidative metabolism, along with a high rate of glutamine oxidation relative to SF188s cells; and 3) the mitochondrial proton leak-linked respiration of SF188f cells increased significantly compared to SF188s cells. It is plausible that the proton leak of SF188f cells may play a role in allowing continuous glutamine-fueled anaplerotic TCA cycle flux by partially uncoupling the TCA cycle from oxidative phosphorylation. Taken together, these rapid, sensitive and high-throughput substrate flux analysis methods introduce highly valuable approaches for developing a greater understanding of genetic and epigenetic pathways that regulate cellular metabolism, and the development of therapies that target cancer metabolism.

## Introduction

Cancer cells significantly reprogram their metabolism to drive tumor growth and survival. Otto Warburg first observed that under aerobic conditions, tumors had high rates of glycolysis compared to the surrounding tissue, a phenomenon known as the Warburg effect, or aerobic glycolysis [1]. He postulated that increased glycolysis and impaired mitochondria respiration is the prime cause of cancer [2]. More recently, a large body of evidence indicates that cancer cells undergo metabolic reprogramming, leading to extensive use of and dependence upon glucose or glutamine for their growth and survival [3]–[9]. This metabolic reprogramming has been shown to be the result of oncogene activation and/or loss of tumor suppressor functions, as well as in response to environmental cues, all of which regulate nutrient substrate uptake and metabolism [10]–[14]. Depending on the combinations of these factors and a given cellular context, cancer cells can manifest an array of metabolic phenotypes [15] , which may impact either treatment selection or response to treatment.

In view of numerous types of genetically and metabolically diverse cancer cells, a rapid, informative, relatively easy-to-perform and higher-throughput substrate flux analysis can facilitate greater understanding of the genetic and epigenetic pathways that regulate cancer cell metabolism, determining whether there is a finite number of metabolic phenotypes among all type of cancer cells, independent of tissue origin, and discovering agents that target specific metabolic pathways for cancer treatment.

Cells produce ATP via two major energy-producing pathways: glycolysis and oxidative phosphorylation. The glycolytic pathway converts glucose to pyruvate. One fate of the pyruvate is reduction to lactate in the cytosol in an oxygen-independent biochemical reaction resulting in ATP production and net proton production. Protons are pumped out of the cell by various mechanisms to maintain the intracellular pH [16] and the efflux of the protons into the extracellular space or medium surrounding the cells causes extracellular acidification [17]–[21]. The major nutrient substrates glucose, glutamine, and fatty acids can be completely oxidized to into CO_2_ and H_2_O via the tricarboxylic acid cycle (TCA cycle) which requires the electron transport chain (ETC) in the mitochondria using oxygen as a terminal electron acceptor, and which is coupled to ATP production by oxidative phosphorylation. The CO_2_ produced can be converted to bicarbonate and protons as catalyzed by carbolic anhydrase [16], another source of protons causing medium acidification. In many non-transformed differentiated cells such as neurons, oxidative phosphorylation produces most of the cellular ATP. In contrast, cancer cells rely heavily on glycolysis in addition to oxidative phosphorylation for their ATP production [22]. As well as fueling ATP production, glucose and glutamine are essential carbon sources that provide anabolic precursors, some of which (e.g., citrate and oxaloacetate) are produced through a truncated TCA cycle for the biosynthesis of lipids, nucleic acids and amino acids.

Since living cells do not store ATP, they produce it continuously and on demand, and therefore constantly consume oxygen and fuel substrates. Thus, the demand for ATP in cells (i.e. ADP availability) controls the rate of oxygen consumption. Electrons (energy) stored in nutrient substrates are extracted via the mitochondrial TCA cycle reactions and carried by reduced electron carriers NADH and FADH_2_ to the ETC. As the electrons flow down the ETC, the energy released is used to pump protons from the matrix into the intramembrane space, forming a transmembrane electrochemical proton gradient across the mitochondrial inner membrane. At the end of the ETC, the electrons are transferred to molecular oxygen, reducing it to water via the terminal cytochrome C oxidase. As protons return to the mitochondrial matrix through the ATP synthase complex, the energy stored in the proton gradient then drives the phosphorylation of ADP to ATP coupling respiration (electron transport) with ATP production. Oxidative phosphorylation, however, is incompletely coupled to respiration. Protons can also re-enter the matrix via proton channels such as uncoupling proteins (UCP), which are located on the inner membrane, bypassing ATP synthase and dissipating the energy gradient without producing ATP in a process known as proton leak. Partially reduced oxygen species (ROS) such as the superoxide anion can be produced at different sites in the ETC depending on conditions [23]; the proton leak has been considered an important cellular mechanism for protecting cells from oxidative damage through lowering ROS produced by the ETC [24].

Given the connection between oxygen consumption and extracellular acidification with nutrient substrate metabolism, increased oxygen consumption is a measure of substrate oxidation when a substrate is added to cells. Likewise, an increase in the rate of extracellular acidification upon glucose addition is a measure of glycolytic flux.

Various traditional experimental methods that analyze substrate metabolism have contributed to the current understanding of cellular metabolism. These include measuring the accumulation of radio-labeled end products such as H_2_O and CO_2_ metabolized from substrates such as ^3^H labeled glucose and ^14^C labeled fatty acids. Other previously used (and more recently developed) methods to quantify metabolites are stable isotope tracers coupled with mass-spectrometry and NMR analysis, both of which have yielded detailed information on substrate metabolism. These techniques, however, can nevertheless be either labor intensive and cumbersome, and/or relatively inaccessible for many laboratories.

This study had two main objectives. The first was to apply the principles described above to establish a series of rapid and easy methods to analyze glycolytic flux and oxidative substrate flux of cancer cells. This was achieved by measuring cellular oxygen consumption rate and extracellular acidification using the XF Extracellular Flux analyzer [22]. The second was to perform proof-of-principle experiments through applying these substrate flux methods, along with analyzing mitochondrial bioenergetics in human glioblastoma cells SF188s and SF188f to interrogate their metabolic networks.

## Materials and Methods

### Reagents

Carbonyl cyanide 4-(trifluoromethoxy)phenylhydrazone (FCCP), myxothiazol, antimycin A, rotenone, 2-deoxyglucose, oxamate, aminooxyacetate, glucose, sodium pyruvate and sodium palmitate were obtained from Sigma (St. Louis, MO, USA). L-glutamine was obtained from Invitrogen (Carlsbad, CA). Oligomycin was obtained from EMD (San Diego, CA, USA). Bovine Serum Albumin fraction V (fatty acid ultra-free) was obtained from Roche Diagnostics (Indianapolis, IN, USA). All compounds and medium were prepared according to the manufacturers' instructions unless indicated otherwise.

### Cell lines and cell culture

Human glioblastoma SF188 cells were obtained from the University of California at San Francisco Brain Tissue Bank. These cells were originally maintained, as recommended by the originator, in an MEM medium containing 5.5 mM glucose and 2 mM L-glutamine. They were adapted step-wise to DMEM medium containing 25 mM glucose and 6 mM L-glutamine as a model system for the study of glutamine metabolism as reported [6]. As a control, the parental cells were also adapted in parallel to DMEM medium containing 5.5 mM glucose and 2 mM L-glutamine. The former acquired a much more rapid growth rate after 4 weeks culture in the medium and was named SF188f (fast). The latter, however, maintained similar growth rates as those parental cells maintained in MEM, and were named SF188s (slow). To maintain their distinct growth phenotype, SF188s and SF188f cells were always cultured in DMEM containing 5.5 mM glucose and 2 mM L-glutamine and 25 mM glucose and 6 mM L-glutamine, respectively at 37°C in a Forma incubator with 10% CO_2_ and 100% humidity at ∼80% confluence in 175 cm^2^ T-flasks (Corning). Human prostate cancer PC-3 and cervical cancer HeLa cells were purchased from American Type Culture Collection (Manassas, VA, USA), and were maintained in RMPI1640. All cell culture media were supplemented with 10% fetal bovine albumin (FBS, Hyclone, Logan, UT, USA).

### XF assay medium

A base medium was used for the assays described in this study directly or supplemented with substrates and cofactors as specified in each of the specific assays (see below) and for each experiment. The base assay medium was prepared as follows. Dulbecco's Modified Eagle's Medium (DMEM) powder (Sigma, catalog number D5030) was the starting material. It contains no glucose, L-glutamine, sodium pyruvate, sodium bicarbonate, or phenol red, and has a low phosphate (see Materials S1 in File S1for details of preparation). In addition, a modified Krebs-Henseleit- bicarbonate buffer (KHB) which contains no bicarbonate and lower phosphate can also be used (Materials S2 in File S1) as an alternative assay medium for fatty acid oxidation. The use of amino acid-free buffer, as opposed to the base medium for other assays is possible but, may result in different experimental outcomes which may require different data interpretations. All the assays described here were performed solely in the base medium with indicated supplementation, with the exception of fatty acid oxidation which was performed in both base medium and KHB, yielding similar results.

### Preparation of palmitate-BSA conjugate

Sodium palmitate was solubilized by warming it to 68°C in 150 mM sodium chloride solution. It was then bound to BSA in solution at molar ratio of 6∶1. The complete protocol is described in Material S3 in File S1.

### Measurement of oxygen consumption rate and extracellular acidification rates

OCR and ECAR measurements were performed using the XF24 or XF96 Extracellular Flux analyzer (Seahorse Bioscience, North Billerica, MA) as described [22]. Briefly, cells were plated into XF24 (V7) or XF96 (V3) polystyrene cell culture plates (Seahorse Bioscience, North Billerica). SF188s cells were seeded at 30,000/well (XF24 plate) or 20,000/well (XF96 plate) and SF188f cells at 20,000/well (XF24 plate) or 15,000/well (XF96 plate), respectively. PC-3 and HeLa cells were plated at 25,000 and 30,000 cells per well, respectively, in XF24 cell culture plates. The cells were incubated for 24 to 28 hours in a humidified 37°C incubator with 10% CO_2_ (DMEM medium) or 5% CO_2_ (RMPI1640 and MEM medium), respectively. Because the two cell lines proliferate at different rates during the 24–28 hour incubation period, SF188s and SF188f cells were treated with trypsin and then counted to determine the cell number in each well after an assay. These cell counts were used to normalize either OCR or ECAR. Their viabilities, as determined after assays, were nearly indistinguishable regardless of the presence or absence of exogenous substrates or metabolic inhibitors in the assay medium. Prior to performing an assay, growth medium in the wells of an XF cell plate was exchanged with the appropriate assay medium to achieve a minimum of 1∶1000 dilution of growth medium. 600 µL (XF24) or 150 µL (XF96) of the assay medium was added to cells for an XF assay. While sensor cartridges were calibrated, cell plates were incubated in a 37°C/non-CO_2_ incubator for 60 minutes prior to the start of an assay. All experiments were performed at 37°C. Each measurement cycle consisted of a mixing time of 3 minutes and a data acquisition period of 3 minutes (13 data points) for the XF24, and 2 min and 4 min for the XF96. OCR and ECAR data points refer to the average rates during the measurement cycles. All compounds were prepared at appropriate concentrations in desired assay medium and adjusted to pH 7.4. A volume of 75 µL for XF24 (25 µL for XF96) of compound was added to each injection port. In a typical experiment, 3 baseline measurements were taken prior to the addition of any compound, and 3 response measurements were taken after the addition of each compound. OCR and ECAR were reported as absolute rates (pmoles/min for OCR and mpH/min for ECAR) or normalized against cell counts, or expressed as a percentage of the baseline oxygen consumption. In this study, baseline OCR or ECAR (a technical term) refers to the starting rates prior to the addition of an agent, which can be used for comparisons with those rates after the addition. In contrast, basal OCR or ECAR (a biological term) refers to OCR or ECAR which occur in cells at rest in order to maintain basic cell function. Unless otherwise specified, the third measurement of baseline or after addition of each substrate or compound was used to generate absolute OCR or ECAR values. As well, percentage of baseline OCR values was calculated as OCR at the third measurement after an agent injection divided by the OCR immediately before the injection. Each datum was determined minimally in triplicate.

### XF Substrate Flux Assay Conditions

#### Glycolytic flux and glycolytic capacity

The assay medium consists of the base medium supplemented with 2 mM L-glutamine. Glutamine is required to achieve the maximal glycolysis rate for some, but not all cell lines. The same medium was used to determine glycolytic capacity. The concentration of glucose added to initiate glycolysis and measure glycolytic capacity was 10 mM, greater than the saturation point under both conditions.

#### Glucose oxidation

The assay medium was the base medium without any exogenous fuel substrate supplementation. The concentration of glucose added to initiate glucose oxidation was 10 mM, which was determined in preliminary experiments to be above saturation.

#### Glutamine oxidation

The assay medium was the base medium without any exogenous fuel substrate. The concentration of glutamine added to the cells to initiate glutamine oxidation was 4 mM, which was also pre-determined to be above saturation.

#### Fatty acid oxidation

The assay medium was the base medium (or KHB ) supplemented with 5.5 mM glucose and 50 µM carnitine (required to transport long chain fatty acid into the mitochondria). Fatty acids tested include long chain fatty acid palmitate, medium chain fatty acid octanoate, and short chain fatty acid butyrate. They were titrated for concentrations stimulating maximal OCR response. The working concentration of palmitate conjugated with BSA was 150 µM, and octanoate 1 mM, which were also above saturation.

It is critical that the above assay conditions are strictly adhered to. Any variation in the assay medium composition may result in different interpretations and insights into cellular metabolic network. Saturating substrate concentrations and optimal compound concentrations were determined by performing titration experiments as described in Materials S4 and S5 in File S1. The effect of assay conditions on the interpretation of experimental results will be described elsewhere.

### Cell counts

SF188s and SF188f cells were detached with trypsin-EDTA and harvested immediately following an XF assay. The number of cells in each well was determined using a ViCell automated trypan blue counter (Beckman-Coulter, Fullerton, CA), and was used to normalize OCR and ECAR as indicated in the figure legends.

### Statistical analysis

The data were presented as mean ± standard deviation, with at least three replicates used for each data point. Unless otherwise indicated, a paired Student's *t* test was performed for each experimental group to assess the statistical significance against respective controls.

## Results

### Glycolysis and glycolytic capacity

**Figure 1:**
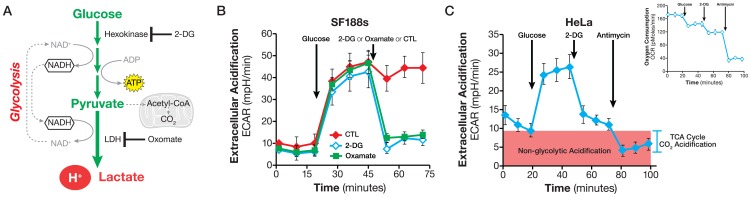
Analyzing Glycolytic flux. A. Schematic illustration of the glycolytic pathway. NADH produced in the cytosol as glucose is converted to pyruvate and is regenerated by LDH in the cytosol. B. Kinetic ECAR response of SF188s cells to glucose (10 mM) and 2-DG (100 mM) or oxamate (100 mM), respectively. SF188s cells were plated at 30,000 cells/well in XF24 V7 cell culture plates 24–28 hours prior to the assays. The assay medium was substrate-free base medium (as described in Material and Methods) supplemented with 2 mM glutamine. The ECAR value was not normalized. A representative experiment out of at least three is shown here. Each data point represents mean ± SD, n = 4. C. ECAR response of HeLa cells to glucose (10 mM), 2-DG (100 mM) and antimycin (1 µM). Insert: the OCR response in the same experiments showing the Crabtree effect and that glucose did not increase OCR. HeLa cells were plated at 30,000/well in XF24 cell culture plates 24–28 hours prior to the assays. ECAR or OCR values were not normalized. The assay medium was substrate-free base medium (as described in Material and Methods) supplemented with 2 mM glutamine. A representative experiment out of at least three is shown here. Each data point represents mean ± SD, n = 5.

We have previously shown that glycolysis accounts for ∼80% of total ECAR in a number of cancer cells as determined through two methods: a) removing glucose from the assay medium and b) adding glycolytic pathway inhibitors such as hexokinase inhibitor 2-DG and lactate dehydrogenase (LDH) inhibitor oxamate [22]. The remaining 20% of the ECAR can be attributed to other metabolic processes, such as the TCA cycle CO_2_ evolution. In order to measure glycolysis using ECAR more accurately and easily, we took the following approach. Glucose is added to cells that are incubated in a glucose-free medium, but supplemented with glutamine (see Materials and Methods). The ECAR increase following the addition of glucose establishes the glycolysis rate. A subsequent addition of a glycolysis inhibitor eliminates the glucose-induced ECAR increase. Any acidification due to other metabolic processes such as the TCA cycle CO_2_ release (from any substrate but glucose) is detected as ECAR prior to glucose addition. The OCR response to glucose, monitored concurrently with ECAR, serves as an indicator of whether glucose is also catabolized through mitochondrial respiration (Figure 1A).

We performed the experiment using SF188s and HeLa cells. As shown in Figure 1B, glucose addition to SF188s cells triggered an instant ECAR increase, 38±4 mpH/min (ECAR of measurement 6 less that of measurement 3), which was subsequently abolished by the addition of glycolysis inhibitors, either 2-DG or oxamate. This experiment indicated that exogenously added glucose was broken down to lactate (because LDH inhibition by oxamate reduces ECAR similarly to 2-DG), causing an ECAR increase and thereby validating our experimental design. Similar results were obtained in HeLa cells (Fig. 1C) which were consistent with our previous study [22], as well as with a number of recent reports showing that the ECAR response parallels that of lactate production [25]–[27]. Prior to adding glucose to the cells as well as following the addition of 2-DG or oxamate, we again observed a small ECAR, 6±1 mpH/min and 10±2 mpH/min (at measurement 3), respectively in SF188s and HeLa cells (Figure 1B and C). We refer to this small but measurable ECAR as non-glycolytic acidification. The OCR response indicated that the injection of glucose not only failed to trigger an increase, but in fact caused a slight decrease in OCR (Figure 1C), which is similar to the Crabtree Effect, first observed in tumor cells by Crabtree in the 1920s [28].

The two most significant proton sources that can contribute to non-glycolytic acidification are the TCA cycle and breakdown of intracellular glycogen, i.e., glycogenolysis [20]. To determine the contribution of the TCA cycle CO_2_ evolution, we used a complex III inhibitor antimycin to stop the electron flow and thus the TCA cycle flux in HeLa cells. Glucose was added first to initiate glycolysis, followed by 2-DG to abolish it, leaving behind non-glycolytic ECAR. The final addition of antimycin stopped the TCA cycle from producing CO_2_. Although a residue remained, 4±1.4 mpH/min, antimycin eliminated about half of the non-glycolytic ECAR (Figure 1C), confirming the contribution of TCA cycle CO_2_-derived proton to non-glycolytic acidification. We tested whether the antimycin-resistent ECAR was due to glycogenolysis by using CP91149, an inhibitor of glycogen phosphorylase (the first enzyme of glycogen breakdown), but we did not observe any significant effect of CP91149 on non-glycolytic acidification (data not shown), leading us to conclude that the residual non-glycolytic ECAR had to be accounted for by other metabolic processes, such as decarboxylation reactions catalyzed by glucose-6 phosphate dehydrogenase and/or pyruvate dehydrogenase. Collectively, these results again confirmed that glycolysis accounts for the majority of ECAR observed in cancer cells, and established that TCA-derived CO_2_ is a primary contributor of non-glycolytic acidification.

Glycolytic flux determined *in vitro* at ambient oxygen levels reflects basal glycolysis rate. When cells experience loss of mitochondrial ATP production due to inhibition of oxidative phosphorylation, either at low oxygen tension or by oligomycin, they augment their glycolytic flux and make more ATP from glycolytic pathways to maintain cellular ATP homeostasis [22]. We refer to this increased glycolytic flux in response to deficiency in mitochondrial ATP production as glycolytic capacity. Experimentally, we define glycolytic capacity as the glucose-induced ECAR by mitochondrial ATP synthase inhibitor oligomycin.

**Figure 2:**
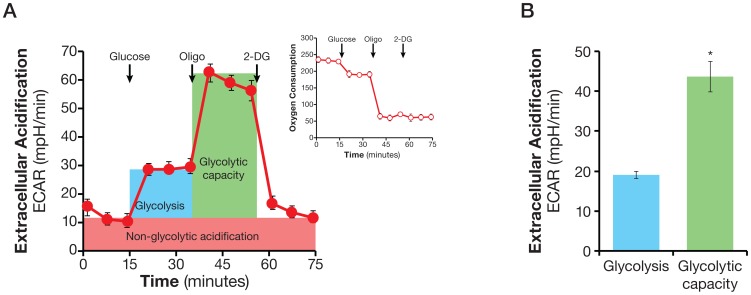
Assaying glycolytic flux and glycolytic capacity. A. Kinetic ECAR response of HeLa cells to glucose (10 mM), oligomycin (2 µM), and 2-DG (100 mM). Insert shows OCR response in response to glucose and oligomycin. HeLa cells were plated at 30,000/well in XF24 V7 cell culture plates 24–28 hours prior to the assays. The assay medium was the substrate-free base medium supplemented with 2 mM glutamine. ECAR or OCR values were not normalized. A representative experiment out of at 5 is shown here. Each data point represents mean ± SD, n = 4. B. Calculated glycolytic flux, glycolytic capacity. Glycolytic flux is the difference between the ECARs of measurement 6 and measurement 3. Likewise, glycolytic capacity describes the difference between the ECAR of measurement 9 and that of measurement 3. * p<0.05.

To determine both glycolytic flux and glycolytic capacity of the same cell population in one experiment, we measured ECAR while consecutively injecting glucose, oligomycin, and 2-DG. As shown in Figure 2A, adding glucose to HeLa cells, as expected, triggered a glycolytic flux of 19±0.9 mpH/min (EACR at measurement 6 less that at measurement 3) in HeLa cells. The subsequent addition of oligomycin caused a further increase in ECAR to 44±3.8 mpH/min (ECAR at measurement 9 less that at measurement 3), indicating an elevated glucose flux toward lactate and revealing the glycolytic capacity of HeLa cells. The final addition of glycolysis inhibitor 2-DG abolished the overall glycolysis (Figure 2A). The calculated glycolytic flux and glycolytic capacity from the glycolysis experiment are shown in Figure 2B.

Two experimental conditions must be met in the above experiment. First, the oligomycin concentration should maximally inhibit respiration. This was achieved by selecting the oligomycin concentration that resulted in maximal OCR inhibition in a titration experiment. For example, 0.5 µM oligomycin was found to be sufficient to achieve maximal inhibition of OCR in HeLa cells (data not shown). Second, it is critical to ensure that the supply of exogenous glucose is saturating, allowing the glycolytic machinery to be the limiting factor. The saturating concentration of glucose for achieving maximal ECAR response was determined in a glucose concentration titration experiment, in which increasing concentrations of glucose were added to the cells, followed by the addition of control or oligomycin at the concentration that maximally inhibits respiration. In HeLa cells, for instance, we found that ECAR increased continuously until the glucose concentration reached 5 mM, after which there was no further ECAR increase (Figure S1). Similar results were obtained with a dozen transformed and non-transformed cell lines (data not shown). We chose a higher-than-saturating concentration of 10 mM as the standard concentration to determine both glycolytic flux and glycolytic capacity.

### Glucose oxidation

**Figure 3:**
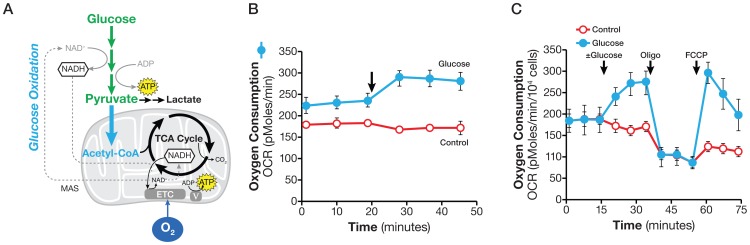
Assaying glucose oxidation. A. Schematic illustration of biochemical pathway for glucose oxidation. The NADH produced in the cytosol as glucose is converted to pyruvate is imported into the mitochondria via the malate-aspartate shuttle and regenerated via the ETC to maintain continuous glucose oxidation. B. Kinetic OCR response of PC-3 cells to glucose (10 mM); C. OCR response to glucose (10 mM), oligomycin (1 µM) and FCCP (0.3 µM). PC-3 cells were plated at 25,000/well in XF24 V7 culture plates. The assay medium was the substrate-free base medium. The OCR values were not normalized. A representative experiment out of three is shown here. Each data point represents mean ± SD, n = 4.

Glucose-derived pyruvate can also enter the mitochondria, where it is converted to acetyl CoA by pyruvate dehydrogenase and enters the TCA cycle via citrate synthase (or as oxaloacetate via pyruvate carboxylase [29]. The acetyl moiety is eventually oxidized to CO_2_ and H_2_O (Figure 3A). The oxygen-consuming process of glucose oxidation first to pyruvate and then to CO_2_ and H_2_O is referred to here as glucose oxidation.

Experimentally, we used the glucose-induced OCR to measure glucose oxidation. In order to establish the glucose oxidation assay, we chose PC-3 cells which actively oxidize glucose. To determine glucose oxidation, 10 mM glucose was added to the cells in assay medium containing no glucose or glutamine (see Material and Methods). As shown in Figure 3B, the addition of glucose to PC-3 cells caused an immediate increase in OCR, 45±11 pmol/min (OCR at measurement 6 less that at measurement 3), indicating glucose flux into the TCA cycle, and ultimately, the ETC for complete oxidation. This experimental design provided a quantitative measurement of glucose oxidation under this experimental condition. In a variant design, PC-3 cells were exposed to glucose, oligomycin, and FCCP consecutively, with 3 measurements before each compound addition and following each compound addition. FCCP uncouples respiration from oxidative phosphorylation, allowing oxidation of any oxidizable substrate present in the assay medium to occur. As shown in Figure 3C, FCCP stimulated a spike in OCR following glucose addition, but not the control, further confirming that the biochemical pathways for glucose oxidation were active in PC-3 cells. The OCR response to FCCP confirms and provides a semi-quantitative assessment of cells' ability to oxidize glucose. In this experimental design, cells were pre-incubated in substrate-free base medium for 60 min prior to an assay, so there is a possibility that they may have been stressed and altered their response to glucose addition. However, this seems unlikely, as OCRs obtained from cells pre-incubated in substrate-free medium and given glucose during the assay were indistinguishable from those of cells that had been pre-incubated with glucose supplemented base medium and were not under the same stress.

### Glutamine oxidation

**Figure 4:**
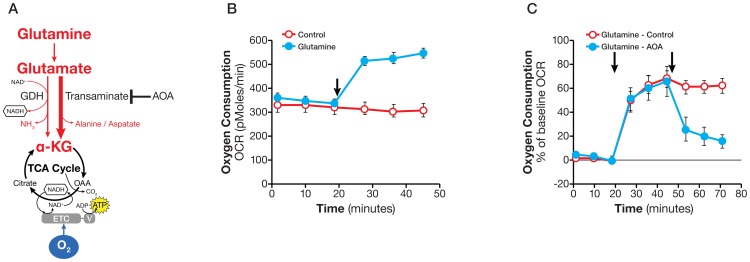
Assaying glutamine oxidation and demonstrating transaminase pathway activity. A. Schematic illustration of biochemical pathway for glutamine oxidation in the mitochondria. B. Kinetic OCR response in SF188f cells to glutamine (4 mM). SF188f cells were plated at 20,000 cells/well in XF24 cell culture plates 24–28 hours prior to the assays. The assay medium was the substrate-free base medium. The OCR value was not normalized. A representative experiment out of three is shown here. Each data point represents mean ± SD, n = 4. C. OCR response (% of baseline) in SF188f cells to glutamine (4 mM) and AOA (100 µM). Glutamine-induced OCR reached 60% over the baseline (OCR at measurement 6 divided by that at measurement 3) while AOA addition reduced it to 20% (OCR at Measurement 9 divided by measurement 3). SF188f cells were plated at 20,000 cells/well in XF24 V7 cell culture plates 24–28 hours before the assays. The % OCR was plotted using measurement 3 as the baseline. The assay medium was the substrate-free base medium. A representative experiment out of three is shown here. Each data point represents mean ± SD, n = 4.

As illustrated in Figure 4A, glutamine enters the mitochondria and is converted to CO_2_ and H_2_O in an oxygen-consuming process, which we refer to here as glutamine oxidation. Glutamine can also be partially oxidized to malate via the TCA cycle, which then exits the mitochondria and is converted to pyruvate by malic enzyme in the cytosol, a process known as glutaminolysis. For simplicity, we refer to both processes as glutamine oxidation.

To measure glutamine oxidation, glutamine was injected into cells in assay medium containing no glutamine (see Material and Methods). We selected SF188f cells which exhibit robust glutamine oxidation to set up the assay. As shown in Figure 4B, the addition of 4 mM glutamine to SF188f cells caused a large increase in OCR, 207±11 pmoles/min (OCR at measurement 6 less that at measurement 3) revealing a high rate of glutamine oxidation. Using the transaminase inhibitor aminooxyacetate (AOA), we determined whether transaminase or glutamate dehydrogenase (GDH) (Figure 4A) was responsible for glutamine oxidation. The addition of AOA following glutamine to SF188f cells abolished glutamine-induced OCR to 20% over the baseline OCR from 60% before the addition of AOA (Figure 4C), suggesting that transaminase-catalyzed α-KG conversion is the major pathway for glutamine's entry into the TCA cycle (Figure 4A). It follows that the remaining AOA-resistant OCR can be attributed to the alternative reaction catalyzed by glutamate dehydrogenase. In an attempt to confirm the GDH-driven glutamine oxidation, we used EGCG, a known GDH inhibitor. Unfortunately, EGCG inhibited OCR regardless of glutamine's presence, suggesting it does not specifically inhibit GDH in this experimental setting, and we were thus unable to confirm GDH-mediated glutamine oxidation. These experiments, however, did allow us to a) measure the glutamine oxidation rate and b) gain insight into the alternate biochemical reactions used for glutamine oxidation. To accurately measure the contribution of transaminase and GDH-to glutamine oxidation, however, additional approaches are necessary. Finally, similar to what observed in glucose oxidation experiment OCRs obtained from cells pre-incubated in substrate-free medium and given glutamine during the assay were indistinguishable from those of cells that had been pre-incubated with glutamine supplemented base medium.

We noticed that a substantial rate of oxygen consumption, 338±8 pmoles/min (measurement 3), occurred in the absence of any exogenously added substrate in SF188f cells prior to glutamine addition (Figure 4B). Similarly, PC-3 cells showed considerable OCR, 234±14 pmoles/min (measurement 3), prior to glucose addition (Figure 3B). This phenomenon is not specific to SF188f or PC-3 cells but is commonly observed, to various extents in a variety of cell lines that we have studied (data not shown). This oxygen consumption in the absence of exogenous substrate is most likely fueled by the oxidation of endogenous substrates; therefore, cancer cells appear to have a significant pool of endogenous oxidizable substrates including fatty acids, amino acids, and glycogen.

### Fatty acid oxidation

**Figure 5:**
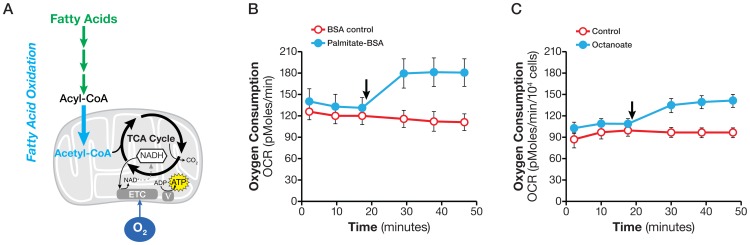
Determining fatty acid oxidation. A. Schematic illustration of biochemical pathway of fatty acid oxidation. B. Kinetic OCR response of SF188f cells to palmitate (150 µM). C. Kinetic OCR response in SF188f cells to octanoate (1 mM). SF188f cells were plated at 15,000 cells/well in XF96 V3 cell culture plates 24–28 hours prior to the assays. Assay medium was the substrate-free base medium supplemented with 5.5 mM glucose and 50 µM carnitine. The OCR value was not normalized. A representative experiment out of four is shown here. Each data point represents mean ± SD, n = 6.

Fatty acids are an important energy source for meeting a high energy demand and maintaining cellular functions in many cells, such as skeletal and cardio muscle cells. Fatty acid oxidation in cancer cells has also been reported but its relevance remains to be fully understood [30]–[34]. Exogenous fatty acids are taken up by cells, converted to fatty acyl CoA in the cytosol, then converted to acetyl CoA in the mitochondria via β–oxidation and ultimately broken down to CO_2_ and H_2_O (Figure 5A). The process of biochemical conversion of fatty acids to CO_2_ and H_2_O consuming oxygen is referred to here as fatty acid oxidation (FAO).

We used increased OCR following injection of exogenously fatty acids to measure the oxidation of fatty acids of various chain lengths, including long, medium and short chains. SF188f cells, which effectively oxidize fatty acids, were chosen to develop the fatty acid oxidation assay. First, we tested the oxidation of the long-chain fatty acid palmitate. Figure 5B shows that the addition of 150 µ M palmitate [8 Acetyl equivalents) to SF188f cells triggered an immediate increase in OCR of 49±5 pmole/min (measurement 7 less measurement 3). Next, we measured oxidation of the medium-chain fatty acid octanoate. The addition of 1 mM octanoate (4 Acetyl equivalents) also increased OCR by 33±4 pmole/min (Figure 5C). Finally, we tested the oxidization of short-chain fatty acid butyrate (2 Acetyl equivalents). The addition of butyrate induced a small but consistent OCR increase (Figure S2). In short, we found that fatty acids of all chains lengths were oxidized by SF188 cells.

### A Shift to oxidative metabolism fueled by glutamine oxidation in rapidly proliferating SF188f glioblastoma cells

**Figure 6:**
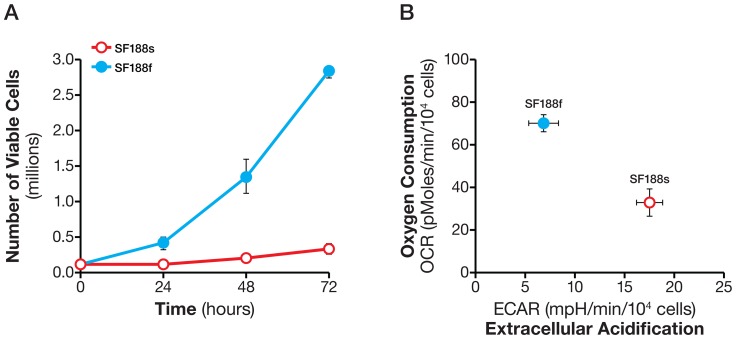
High proliferation rates of SF188f cells and associated shift in their basal OCR and ECAR compared with SF188s cells. A. Number of SF188s and SF188f cells at 24, 48 and 72 hours in their respective culture medium. B. Basal OCR and ECAR of SF188s and SF188f cells. SF188s and SF188f cells were plated at 30,000 and 20,000 cells/well, respectively, in XF24 V7 cell culture plates 24–28 hours prior to the assays. Upon completion of an assay, cells were treated with trypsin and counted for the purpose of normalization; Assay medium was the substrate-free base medium supplemented with 25 mM glucose, 6 mM Glutamine and 1 mM pyruvate. A representative experiment out of three is shown here. The OCR and ECAR values were normalized to pmoles/min/10^4^ cells or mpH/min/10^4^cells. Each data point represents mean ± SD, n = 3.

Having established the methods to analyze glycolysis and oxidation of exogenous substrates, we performed proof-of-principle experiments in SF188s and SF188f cells (see Material and Methods). These two cell lines were derived from the same parental cell line SF188 (which harbor c-*MYC* amplification) [35], but proliferated at very different rates, with the former much slower than the latter (Figure 6A). The fast-growing behavior of SF188 cells occurred only after, and not within, the initial four weeks' culture in DMEM containing 25 mM glucose and 6 mM glutamine, whereas SF188 cells cultured in DMEM containing 5.5 mM glucose and 2 mM glutamine maintained the same growth rate as the parental cells, suggesting an intrinsic change in SF188f cells' growth program as they acquired the fast growing behavior. We were curious as to whether there were any metabolic alterations associated with SF188f cells' fast growing behavior. Upon examining their basal OCR and ECAR in assay medium containing 25 mM glucose, 6 mM glutamine and 1 mM pyruvate, we found that SF188f cells displayed a lower ECAR and a higher OCR compared with SF188s cells (after adjusting for the number of cells being measured) (Figure 6B). This suggested that the fast growing SF188f glioblastoma cells adopted a more oxidative metabolism (perhaps reprogramming their metabolic network via certain epigenetic events), shifting away from glycolytic metabolism.

**Figure 7:**
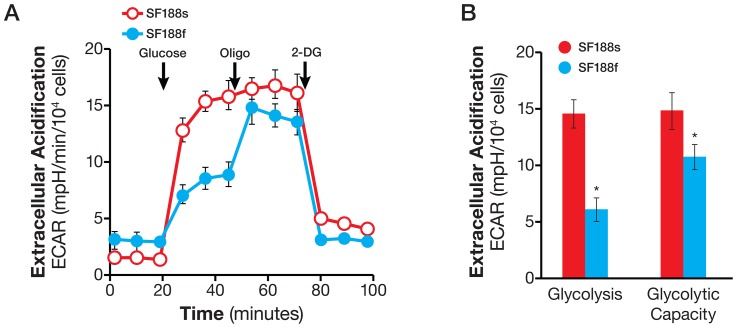
Lowered basal glycolytic flux but acquired glycolytic capacity in SF188f cells compared with SF188s cells. A. ECAR response of SF188s and SF188f cells to glucose (10 mM), oligomycin (1 µM), and 2-DG (100 mM). SF188s and SF188f cells were plated at 30,000 and 20,000 cells/well, respectively, in XF24 V7 cell culture plates 24–28 hours prior to the assays. The assay medium was the substrate-free base medium supplemented with 2 mM glutamine. Upon completion of an assay, cells were treated with trypsin and counted for the purpose of normalization. ECAR values were normalized to mpH/10^4^ cells. A representative experiment out of three is shown here. Each data point represents mean ± SD, n = 4. B. Calculated glycolytic flux and glycolytic capacity of SF188s and SF188f cells normalized to mpH/min/10,000 cells. * p<0.05.

Having determined the basal OCR and ECAR, we investigated glycolytic and oxidative substrate flux in these cells. First, we examined the glycolytic arm of metabolism. The normalized basal glycolytic flux was much lower in SF188f cells, at 5.6±0.8 mpH/10^4^ cells, compared with SF188s cells, at 14.4±0.8 mpH/10^4^ cells (Figure7A). Interestingly, oligomycin stimulated a large ECAR increase over basal glycolysis in SF188f cells (Figure 7A), but failed to evoke a significant increase in SF188s cells. Thus, under basal conditions, glycolysis in SF188s cells occurs at full capacity while SF188f cells possess a substantial unused glycolytic capacity. The respective glycolysis flux and glycolytic capacity of the pair are shown in Figure 7B.

**Figure 8:**
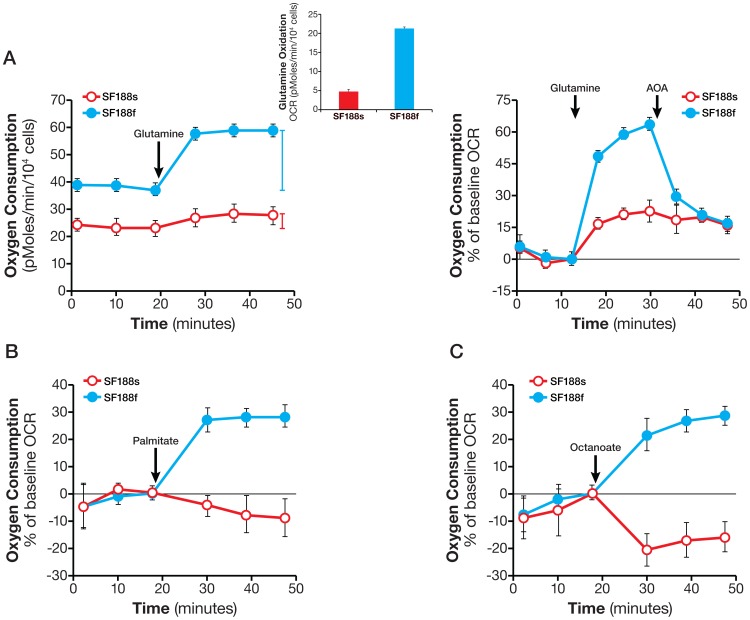
Enhanced glutamine oxidation and activation of fatty acid oxidation in SF188f cells. A. Kinetic OCR response of SF188s and SF188f cells to glutamine (4 mM) (left panel). Insert: calculated glutamine oxidation rate of SF188s and SF188f cells. OCR response (% of baseline) to glutamine and AOA (100 µM) (right panel). SF188s and SF188f cells were plated at 30,000 and 20,000 cells/well, respectively, in XF24 V7 cell culture plates 24–28 hours prior to the assays. The assay medium was the substrate-free base medium. Upon completion of an assay, cells were treated with trypsin and counted for the purpose of normalization. The OCR values were normalized to pmoles/min/10^4^ cells. A representative experiment out of three is shown here. Each data point represents mean ± SD, n = 4. B and C. OCR response of SF188s and SF188f cells to palmitate-BSA (150 mM) (B) and octanoate (1 mM) (C). SF188s and SF188f cells were plated at 20,000 and 15,000 cells/well, respectively, in XF96 V3 cell culture plates 24–28 hours prior to the assays. The assay medium was the substrate-free base medium supplemented with 5.5 mM glucose and 50 µM carnitine. Fatty acid oxidation was expressed as % OCR plotted using measurement 3 as the baseline. A representative experiment out of three is shown here. Each data point represents mean ± SD, n = 6.

Second, we investigated the oxidation of glutamine and fatty acids in the cells. Glutamine oxidation occurred in both, but at a much higher rate in SF188f cells (21±0.3 pmol/min/10^4^ cells) than in SF188s cells (4.6±0.6 pmol/min/10^4^ cells) (Figure 8A). To distinguish the responsible biochemical pathways, we used transaminase inhibitor AOA. As shown in Figure 8A, AOA largely abolished glutamine-stimulated OCR in SF188f cells, but had little effect on SF188s cells. These results suggested that the transaminase pathway in the rapidly dividing SF188f cells was not only activated but became the primary pathway in the oxidation of glutamine. In contrast, the GDH pathway, as inferred from AOA-resistant fraction of glutamine –evoked OCR remained the sole pathway carrying out glutamine oxidation in the slowly-dividing SF188s cells. Similarly, palmitate and octanoate oxidation took place in SF188f cells, but not in the slower-growing SF188s cells (Figure 8B), suggesting SF188f cells had acquired the ability to take up and oxidize both long and medium-chain fatty acids. Collectively, these results indicated that glutamine and fatty acid oxidation was activated as SF188 cells adapted to a nutrient-excessive environment.

Glucose oxidation of SF188s and SF188f cells was also examined. Glucose addition did not stimulate any increase in OCR in either cell line (data not shown) suggesting neither oxidizes glucose.

**Figure 9:**
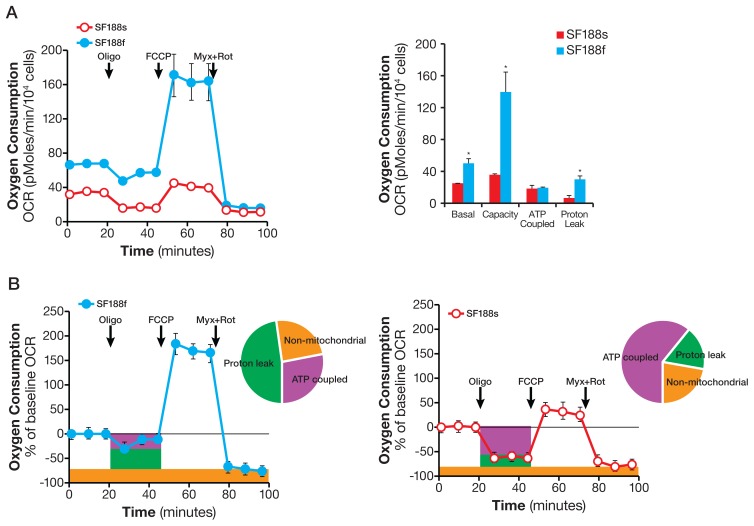
Markedly reprogrammed mitochondrial bioenergetic machinery of SF188s and SF188f cells. A. Kinetic OCR response of SF188s and SF188f cells to oligomycin (1 and 2 µM respectively) to determine ATP coupled respiration, FCCP (0.3 µM) to establish maximal respiratory capacity, and myxothiazol (1 µM) and rotenone (1 µM) cocktail) to define mitochondrial respiration (left). Calculated ATP-coupled respiration, proton leak-linked respiration, maximal mitochondrial respiratory capacity (right). SF188s and SF188f cells were plated at 30,000 and 20,000 cells/well, respectively, in XF24 V7 cell culture plates 24–28 hours prior to the assays. The assay medium was the substrate-free base medium supplemented with 25 mM glucose, 6 mM glutamine and 1 mM pyruvate. Upon completion of an assay, cells were treated with trypsin and counted for the purpose of normalization. OCR values were normalized to pmoles/10^4^ cells. A representative experiment out of three is shown here. Each data point represents mean ± SD, n = 3. *p<0.05. B. Kinetic OCR response (% of baseline, baseline = measurement 3) of SF188f (right) and SF188s cell (left) with distribution of ATP-coupled respiration (% of oligomycin-sensitive at measurement 4) and proton leak-linked respiration (oligomycin-resistant at measurement 4 but myxothiazol-sensitive mitochondrial OCR at measurement 12) and non-mitochondria respiration (myxothiazol-resistant OCR at measurement 12).

Having established differential oxidation of glutamine and fatty acids in SF188s and SF188f cells, we proceeded to investigate the mitochondrial bioenergetics machinery [36], [37] by consecutively adding oligomycin, FCCP, and complex III inhibitor myxothiazol in the presence of saturating amounts of a full set of energy substrates (25 mM glucose, 6 mM glutamine and 1 mM pyruvate for both cell lines). As shown in Figure 9, the mitochondrial bioenergetic state of SF188f differed strikingly from that of SF188s. First of all, in addition to a higher basal OCR, the mitochondrial respiratory capacity (determined by FCCP-stimulated OCR, Figure 9A)) was much higher while the fraction of basal OCR contributing to ATP-coupled respiration (revealed by oligomycin-sensitive OCR) in SF188f cells was greatly attenuated (Figure 9B). Intriguingly, proton leak (the difference between oligomycin-resistant but myxothiazol-sensitive OCR) in SF188f was markedly increased to 48% of the baseline OCR compared with 17% in SF188s cells (Figure 9B). These results suggested that basal respiration in SF188f cells was largely uncoupled from phosphorylation of ADP to ATP.

## Discussion

In this study, we presented the concept, design, and development of several methods to analyze the glycolytic flux and oxidative flux of glucose, glutamine, and fatty acids in cancer cells. In the proof-of- principle experiments using human glioblastoma SF188s and SF188f cells, we provided initial evidence suggesting a mechanism that may allow continuous anaplerotic glutamine flux by increasing mitochondrial proton leak partially uncoupling the TCA cycle from oxidative phosphorylation in the fast proliferating SF188f cells.

### Glycolysis and glycolytic capacity

Extracellular acidification, or ECAR, is comprised of glycolytic and non-glycolytic acidification. The former is the major contributor to total ECAR in cancer cells studied to date, while the latter accounts for significant albeit small fraction, which is mainly due to TCA cycle CO_2_ production (Figure 1). Our experimental design makes it possible to directly identify and calculate glycolytic and non-glycolytic ECAR (Figure 1 and 2). Glucose-derived CO_2_ obviously can contribute to glycolytic ECAR. This can be ruled out by examining the OCR response to glucose in the same experiment. If the addition of glucose does not increase OCR of the cells, or inhibits OCR (the Crabtree effect) as we observed in HeLa cells (Figure 1C and 2A), then there is no glucose-derived CO_2_ in the assay. In fact, most of the cancer cells we studied exhibit the Crabtree effect (data not shown). Thus in this experimental setting, glucose-induced ECAR increase is an accurate measurement of glycolytic flux. In any case where glucose increases the OCR, additional experiments such as the addition of a carbonic anhydrase inhibitor will help ascertain whether glucose-derived CO_2_ is converted to protons at all, and if so, how significant a contribution to ECAR it might be.

The glycolytic capacity of cells under basal conditions upon which cells can draw in the face of increased energy demand, for example, a condition imposed by the loss of mitochondrial ATP, allows cells to maintain energy homeostasis and cellular function. By extension, it can serve as a mechanism by which cancer cells adapt to and survive under hypoxic conditions [8]. A recent study provides evidence in favor of this notion [38]. These authors found that prostate cancer cells PC-3 displayed a significant glycolytic capacity in addition to higher glycolytic flux compared with normal prostate epithelial cells. However, our observation that the glycolytic capacity of glioblastoma SF188s and SF188f cultured under different nutrient condition were either nonexistent or substantial (Figure 7) suggest a more complex story which requires further investigation. The glycolytic flux and glycolytic capacity assay offers a very useful tool for those future studies.

At least three mechanisms can explain glycolytic capacity. In the first, cells can simply augment glucose uptake and/or the activity of glycolytic enzymes. For example, glucose transporters have been shown to relocalize rapidly from the cytoplasm to the cell surface when under energy stress, resulting in an immediate increase in glucose uptake [39]–[41]. In the second mechanism, glucose-derived pyruvate can be redirected away from the mitochondrial TCA cycle to lactate production, when oxidative phosphorylation is suppressed by oligomycin. In the third mechanism, glucose carbons can be shunted away from the production of metabolic intermediates for biosynthesis (including ribose, lipids, and serine synthesis) [13], [42] and toward lactate production. The first two mechanisms most likely account for the increased glycolytic flux (i.e., glycolytic capacity), but exactly which mechanism(s) are responsible for glycolytic capacity may depend on cellular context of the cells under study.

### Oxidative Substrate Flux

To produce ATP via oxidative phosphorylation, all nutrient substrates taken up by cells are ultimately oxidized via the TCA cycle and the ETC. In order to measure substrate oxidation, we applied this theory using the availability of an exogenous substrate (glucose, glutamine and fatty acids) at above saturation concentration to control oxygen consumption (i.e., electron transport). This principle is generally applicable to any metabolic substrate such as pyruvate, ketone bodies, and succinate (unpublished observations). Although being rapid and with higher throughput, these methods evidently do not yield as detailed information as metabolomics does. The overall metabolic insights gained, however, can either guide and/or be complementary to tracer-based metabolomics analysis to quantitatively follow substrate carbon flux through specific metabolic pathways [43].

Two technical points warrant further discussion. First, by selecting an above saturation substrate concentration in the substrate flux assays, the OCR or ECAR values are not governed by the amount of substrate available, but rather by the cells' ability to catabolize each substrate in meeting the demand of cell proliferation and maintaining basic cell functions. This experimental design makes it possible to evaluate substrate metabolism across many cell lines, regardless of the substrate concentrations in their growth media. For example, the growth media for SF188s and SF188f contained 2 and 6 mM glutamine, respectively; however, 4 mM (above saturation) glutamine, was used to compare glutamine oxidation rate of both (Figure 8A). Second, substrate oxidation rates as determined by the acute substrate addition described here are approximations of those in culture conditions (in which serum is present. They do, however, reveal the cells' ability to oxidize the substrates – in other words, the existence of the metabolic programs – providing clues for further investigation using OCR/ECAR and/or other technologies, such as tracing with stable isotope labeled substrates. For example, the effect of serum or growth factors on substrate oxidation can be ascertained by using variations upon the original assays, either by including them in the assay medium or by adding them acutely following substrate addition. Therefore, the substrate oxidation methods presented here can serve as the foundation upon which additional studies can be devised to gain further insights into the dynamic cellular metabolic network.

In this study, we not only determined basal respiration but also identified the type of exogenous nutrient substrates cells are able to oxidize and the rates at which they can be oxidized under the experimental conditions. Our results show that SF188f cells possess the ability to oxidize glutamine at a higher rate compared with SF188s cells. As shown in Figure 8A, OCR increases by ∼20 pmoles/min/10^4^ cells from ∼40 to ∼60 pmoles/min/10^4^ cell following glutamine addition. The glutamine oxidation results obtained here are consistent with previously reported high glutamine utilization in SF188 cells employing classical glutamine consumption method [6]. Interestingly, unlike glutamine and fatty acids, glucose oxidation remained unaltered in SF188f cells. These results again highlight cancer cells' substrate preferences and their dynamic shift in response to environmental alterations, which can be readily revealed by these methods.

In addition to respiring on exogenously supplied substrates, we observed substantial respiration with endogenous substrates in cancer cells; for example, SF188f and PC-3 cells (Figure 3 and 4). As we reported previously, fatty acids are one such type of endogenous substrate [30]. However, oxidation of endogenous substrates is a poorly understood process, as well as its physiological role of in normal or cancer cells. Recent studies showed that macroautophagy and chaperone-mediated autophagy are required to maintain energy metabolism for tumor growth and survival [43], [44]. Linking the observed respiration with endogenous substrates, we speculate that autophagy may, at least in part, provide endogenous substrates in the form of amino acids and fatty acids. The methods established in this study can measure respiration of both exogenous and endogenous substrates, thus affording an excellent opportunity for conducting such studies.

### A potential role of glutamine oxidation along with proton leak in SF188f cells

The high rate of glutamine oxidation observed in SF188f cells was also accompanied by an unusually large proton leak. 48% of the cells' basal respiration was found to be diverted to proton leak, with a smaller fraction of 28% devoted to ATP production (Figure 9). We suggest that this unusually large proton leak, along with high levels of glutamine oxidation, may allow on-demand glutamine anaplerotic flux to meet the biosynthetic demand required for biomass duplication in rapidly dividing SF188f cells. Specifically, proton leak can partially uncouple the TCA cycle flux from mitochondrial ATP production (reducing ATP yield) and thus bypass the control of oxidative phosphorylation exerted on the anaplerotic TCA cycle flux. A similar result which supports our hypothesis has been recently reported in H-RAS transformed mouse embryonic fibroblasts (MEF) [45]. In the H-RAS expressing MEF, an overexpression of kinase suppressor of ras 1 (KSR1), a scaffold protein in the ERK signaling pathway, Leads to significantly increased glutamine oxidation as well as proton leak compared with the KSR1 deficient MEF. Similarly, these metabolic alterations in the H-RAS MEF were also accompanied by faster cell proliferation. Our results provide the initial evidence suggesting a potential role for proton leak in modulating anabolic precursor production in rapidly proliferation SF188f cells. Of course, much more broad and in-depth studies are required to substantiate this possibility.

### Conclusion

**Figure 10:**
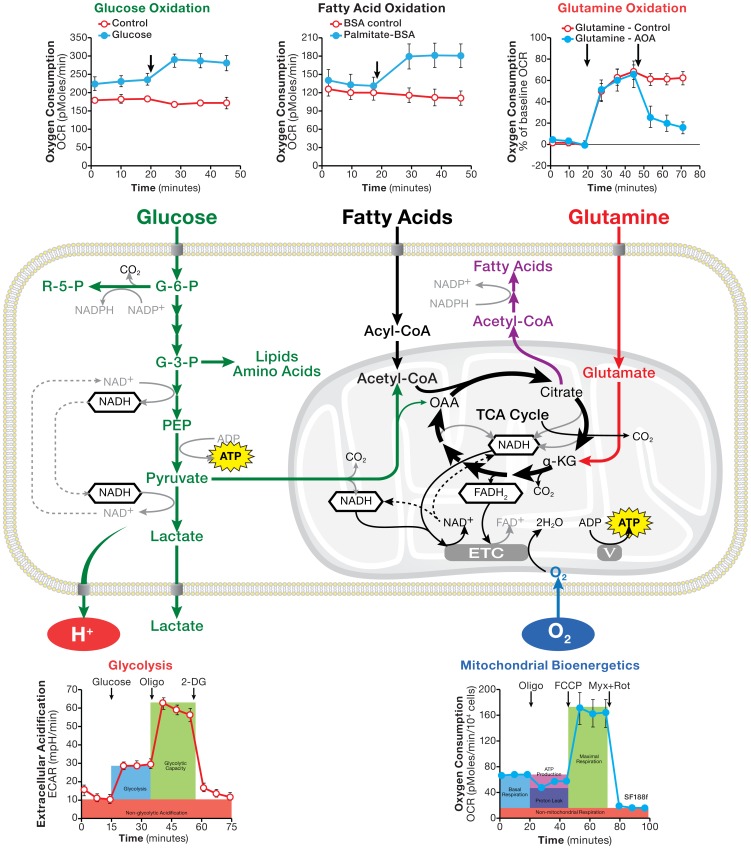
Schematic illustration of cellular metabolism pathways along with assays of glucose, glutamine, and fatty acid oxidation, glycolytic flux and mitochondrial bioenergetics.

By measuring OCR and ECAR responses of living cells to the addition of substrates along with metabolic perturbation agents, the methods presented here have cleared a path for easy and systematic analyses of metabolic substrate flux in wide range of cancer cell lines (Figure 10). These microplate-based substrate flux methods use only small amounts of biological materials, making it possible to analyze large numbers of natural, genetically altered, or compound-treated cancer or non-cancer cell samples in a short period of time. The approach is highly valuable, particularly when integrated with genomic, proteomic, and metabolomics technologies with the purpose of understanding of genetic, epigenetic, and signaling pathways that regulate cancer cell metabolism and cancer cells' metabolic vulnerabilities. Given the growing appreciation of metabolic shifts and nutrient substrate switches in many other diseases, including cardiovascular, neurological and immune ailments, these new methods are also very useful for investigating pathobiology in a wide variety of disease models.

## Supporting Information

Figure S1 **Determining saturating glucose concentration at which maximal ECAR response were achieved under both basal condition and inhibition of oxidative phosphorylation by oligomycin.** A. Glucose at concentrations of 1, 5, 10 and 25 mM were injected sequentially to PC-3 cells. PC-3 cells were plated at 25,000/well in XF24 V7 culture plates. The assay medium was the base medium supplemented with 2 mM L-glutamine. The OCR values were not normalized. A representative experiment out of three is shown here. Each data point represents mean ± SD, n = 4. B. Oligomycin (1 µM) was added to PC3 cells in the same assay medium as in A followed by three sequential injections of glucose at concentrations of 5, 10, and 25 mM as indicated.
(EPS)

Figure S2 **SF188f cells are able to oxidation short chain fatty acid bytyrate.** A. OCR response of SF188s and SF188f cells to butyrate (0.3 mM). SF188s and SF188f cells were plated at 20,000 and 15,000 cells/well, respectively, in XF96 V3 cell culture plates 24–28 hours prior to the assays. The assay medium was the substrate-free base medium supplemented with 5.5 mM glucose and 50 µM carnitine. Fatty acid oxidation was expressed as % OCR and plotted using measurement 3 as the baseline. A representative experiment out of three is shown here. Each data point represents mean ± SD, n = 6.
(EPS)

File S1 **Materials S1-S5.**
(DOCX)
