# Supplementary material for: Rapid Analysis of Glycolytic and Oxidative Substrate Flux of Cancer Cells in a Microplate
Source: PLoS One. 2014 Oct 31;9(10):e109916. doi: 10.1371/journal.pone.0109916 (PMC4215881; doi:10.1371/journal.pone.0109916)
Supplement: File S1 — Materials S1-S5. (DOCX) [file pone.0109916.s003.docx]

**Materials S1 Preparation of base XF assay medium**

To prepare an assay medium, the DMEM powder was dissolved in deionized water (Invitrogen), and additional NaCl was added to a final concentration of 142 mM in total and phenol red to a final concentration of 3 mg/L. The medium was warmed to 37°C before adjusting the pH to 7.4 with sodium hydroxide, and then filtered through a 0.2 μm filter to achieve sterility. This DMEM medium is referred to as XF base assay medium hereafter for measuring OCR and ECAR using the XF Analyzer. The use of bicarbonate-free and low-buffered assay medium must be strictly maintained in order to sustain pH in ambient air and to preserve the sensitivity of the ECAR measurement [[22](#_ENREF_22)]. The XF base assay medium can be supplemented with the desired substrate, such as glucose, glutamine or pyruvate (pertaining to specific experimental designs (see below) and readjusting the pH to 7.4.

In addition to bicarbonate-free and low buffered DMEM XF assay medium, a modified (Krebs-Henseleit- bicarbonate buffer (KHB) that eliminates bicarbonate and lowers phosphate (to 1.8 mM) can also be used as an alternative assay medium, particularly for fatty acid oxidation . It should be noted, however, that amino acids can regulate nutrient substrate uptake. The use of amino acid-free buffer, as opposed to medium may results in different experimental outcomes and may require different data interpretations. All the assays described here were performed in DMEM XF assay medium with the exception of fatty acid oxidation, which was performed in both DMEM and KHB and yielded similar results.

**Materials S2 Preparation of bicarbonate-free KHB buffer**

KHB buffer contains salts in the following concentrations: Sodium Chloride (NaCl) 111mM, Potassium Chloride (KCl) 4.7mM, Magnesium Sulfate (MgSO4) 2mM and Disodium Phosphate (Na2HPO4) 1.2mM). A 5X KHB solution may be prepared and should be diluted in dH2O to 1X prior to use.

**Material S3 Preparation of 1.5 mM palmitate-BSA conjugate (6:1 molar ratio)**

The protocol consists of two steps: prepare a BSA solution and a palmitate solution and subsequent addition of palmitate to BSA for the final conjugation.

1. BSA solution preparation
2. Make 300 mL 150 mM NaCl by adding 9 mL 5 M stock to 291 mL dH2O.
3. Add 3.06 gram ultra-fatty acid free BSA to 90mL 150mM NaCl .
4. Cover with parafilm and place in a pre-warmed 37C water bath on a heated stir plate and stir.
5. Maintain temperature between 37-40˚C, using ice to reduce temperature when necessary
6. Once BSA is completely dissolved, sterile filter.
7. Transfer 50mL filtered BSA to a pre-warmed beaker, cover with parafilm and return to 37˚C water bath and resume stirring.
8. Dilute remaining 40mL BSA with 40 mL 150 mM NaCl for 0.17 mM control BSA stock. Aliquot and store at -20˚C.
9. Sodium palmitate solution preparation and conjugation to BSA
10. Add 45.9 mg of sodium palmitate to 44 ml of 150 mM NaCl solution.
11. Cover with parafilm and place in a second pre-warmed 37C water bath and stir on a heated stir plate.
12. Heat to 70˚C while stirring. Palmitate solution may appear increasingly cloudy as temperature reaches 50-60˚C but will become clear near 70˚C.
13. When Palmitate solution is clear (~70C) transfer 40 mL hot palmitate solution 5mL at a time to the 50 mL of BSA solution stirring at 37˚C.
14. Cover beaker with parafilm and stir at 37˚C for one hour, maintaining water bath temperature between 35-40˚C.
15. Measure volume in 100 mL glass graduated cylinder and adjust to 100 mL with 150 mM NaCl for 1.5 mM palmitate stock.
16. Adjust pH to 7.4, aliquot and freeze conjugated palmitate-BSA at -20˚C.

Note: keeping each solution at the specified temperature is critical for efficient conjugation of palmitate.

**Materials S4 Substrate titration**

In the substrate titration experiments, substrates were prepared at 8, 9, or 10 times of working concentrations. They were then loaded into the reagent ports of a sensor cartridge immediately before an experiment. Glucose was prepared so that the working concentrations, when injected to cells in a well, were 1, 5, 10 and 25 mM for each experimental group. The glucose concentration series were prepared in base assay medium supplemented with 2 mM L-glutamine and without any supplement in glycolysis and glucose oxidation experiment, respectively. L-glutamine was prepared so that the working concentrations reached 1, 2, 4, and 8 mM in each experimental group. The L-glutamine concentration series were prepared in base assay medium without any supplement. Palmitate-BSA conjugate (prepared as above) was used either directly or diluted to reach working concentrations of 100, 150, and 200 µM. Palmitate-BSA was diluted with base assay medium supplemented with 5.5 mM glucose and 50 μM carnitine. Octanoate was prepared in the same medium as palmitate-BSA and brought to working concentrations of 0.25, 0.5, 1 and 2 mM when exposed to cells The concentrations that yielded OCR or ECAR above plateau levels ( i.e. above saturation) were chosen for each assay described in this study.

**Material S5 Compound titration**

Optimal concentrations of oligomycin, antimycin, FCCP and AOA for each relevant cell line were determined by performing serial dilutions of each compound. Oligomycin was prepared in base assay medium supplemented with 2 mM L-glutamine in order to reach working concentrations of 0.125, 0.25, 0.5, 1, 2, and 4 µM when injected into cell wells. Antimycin was prepared in base assay medium for working concentrations of 0.25, 0.5, 1 and 2 µM. FCCP solution was prepared to give final working concentrations of 0.003 0.11, 0.33, 1 and 3 µM. AOA was prepared in base assay medium supplemented with 4 mM L-glutamine to yield working concentrations of 0.25, 0.5, 1 and 2 mM when injected into the cell wells. For oligomycin, antimycin, and AOA, the optimal concentrations chosen were the ones at which OCR inhibition reached a plateau. Due to its biphasic nature, the optimal concentration of FCCP chosen was the one that stimulated peak OCR response.
